# Concussion education in Canadian medical schools: a 5 year follow-up survey

**DOI:** 10.1186/s12909-018-1416-7

**Published:** 2018-12-20

**Authors:** François Mathieu, Michael J. Ellis, Charles H. Tator

**Affiliations:** 1 Division of Neurosurgery, Department of Surgery, University of Toronto, 399 Bathurst St, Toronto, ON M5T2S8 Canada; 2 Pan Am Clinic, Section of Neurosurgery, University of Manitoba, 75 Poseidon Bay, Winnipeg, MB R3M3E4 Canada; 3 Canadian Concussion Center, Division of Neurosurgery, University Health Network, Toronto Western Hospital, 399 Bathurst St, Toronto, ON M5T2S8 Canada

**Keywords:** Concussion, Medical school, Education

## Abstract

**Background:**

Despite concussion now being recognized as a public health priority in Canada, recent studies—including our 2012 survey of Canadian medical schools—have revealed major gaps in concussion education at the undergraduate medical school level.

**Methods:**

We re-surveyed all 17 Canadian medical schools using a questionnaire divided in two categories: (1) concussion-specific education (2) head injury education incorporating a concussion component to determine whether there have been any improvements in concussion education at the medical school level during the last five years. For each year of medical school, respondents were asked to provide the estimated number of hours and teaching format for each category.

**Results:**

We received replies from 13 of the 17 medical schools (76%). 11 of the 13 (85%) medical schools now reported providing concussion-specific education compared to 29% in our 2012 survey. The mean number of hours dedicated to category 1 learning in 2017 was 2.65 h compared to 0.57 in 2012, and the mean number of hours of category 2 increased to 7.5 from 1.54.

**Conclusion:**

Our follow-up study reveals increased exposure to concussion-related teaching in Canadian medical schools during the last five years. Persistent deficiencies in a minority of schools are highlighted. These should be addressed by reiterating the importance of concussion education for undergraduate medical students and by developing clear concussion-specific objectives at the national licensure level.

## Background

Concussion is now recognized as an important public health priority in Canada. A recent national cross-sectional survey estimates its yearly population incidence at 0.29% [1]. Although most concussed individuals recover promptly, up to 30 % experience persistent symptoms, leading to long-term adverse physical and mental health consequences and disability [2, 3]. In order to diagnose a concussion, a clinician must rule out more severe forms of traumatic brain injury, cervical spine injury and other medical and neurological disorders that can present with concussion-like symptoms based on results obtained from a clinical assessment and occasionally diagnostic imaging [4]. Given these requirements, Canadian physicians are uniquely licensed and positioned to play a central role in the diagnosis and management of concussion. However, our 2012 survey identified important gaps in the concussion curriculum in most Canadian medical schools [5]. A similar study performed in the United States highlights persistent deficiencies in concussion-related teaching in American medical schools [6]. This is concerning given that the failure to diagnose and adequately manage concussion can lead to a prolonged recovery such as post-concussion syndrome [7, 8], the potentially fatal second-impact syndrome and neurodegeneration such as chronic traumatic encephalopathy [9]. We therefore designed a follow-up survey to determine whether recent efforts to increase awareness on concussion and growing media coverage of this issue have translated into enhanced concussion education at the undergraduate medical school level.

## Methods

From March 2017 to October 2017, we contacted representatives of all 17 Canadian medical schools. Initial emails containing a description of our study and questionnaire were sent to the Dean or Vice-Dean of undergraduate medical education or designated curriculum manager for each school. A similar format to our 2012 survey was followed: we asked each institution to complete a Concussion Education Questionnaire that consisted of a table with the following questions on curriculum content for each year of medical school: “concussion-specific education” (Yes/No) (Category 1) or “head-injury education incorporating a concussion component” (Yes/No) (Category 2). If yes, we asked the school to estimate the approximate numbers of teaching provided in the form of didactic lectures, problem-based or case-based learning, small group seminars or clinical experiences with direct patient contact.

In addition, we included three follow-up open-ended questions: 1) Have there been any significant changes in the amount or format of teaching with regards to the evaluation and management of concussion since 2011? Please specify 2) If YES, what are the motivating factors for implementing these changes in the curriculum? 3) If NO, can you identify possible reasons or barriers for not incorporating more concussion specific teaching in the curriculum? In addition, space was provided for additional comments deemed relevant by the participating schools. Thematic analysis was used to interpret answers to these follow up questions. Similar to the original survey, we guaranteed school anonymity in the reporting of our results.

We hypothesized that this follow-up survey would demonstrate an increase in the proportion of medical schools offering concussion-related teaching in light of the growing media attention to the topic and recent efforts to establish pan-Canadian management guidelines. Given our small sample size, we used the two-tailed Fisher’s exact test to make comparisons between the proportion of schools offering category 1and 2 teaching between the two study periods.

## Results

13 of the 17 medical schools contacted replied (76%). 11 of the 13 (85%) medical schools reported providing concussion-specific education compared to 29% for our original survey (Table 1, *p*-value< 0.006). All the participating schools offered at least head injury education that incorporated a concussion component (Table 1). Of the 11 schools that participated in both our 2012 and 2017 surveys, 7 (64%) reported an increase in teaching time dedicated to concussion-specific material and 9 (82%) reported an increase in general head-injury education (Table 2). 6 schools that previously did not provide any category 1 education (concussion-specific education) had now incorporated a concussion-specific teaching session to their curriculum. None of the repeat responders reported having eliminated concussion-specific educational sessions. The numbers of total hours specifically dedicated to concussion over the full medical school curriculum ranged from 0 to 8 (mean 2.65 h, SD 2.83) (Table 3 in Appendix). This represents an important increase compared to our 2012 survey (mean 0.57 h, range 0 to 2) (Table 4 in Appendix). The total numbers of hours dedicated to category 2 (head-injury education incorporating a concussion component) materials ranged 1 to 43 (mean 7.5 h) (Table 4 in Appendix). The preferred format for both categories varied considerably between institution as displayed in Table 3.

**Table 1:** Summary of concussion-related education per category (2012 vs 2017)

Survey year	2012	2017	P-value
Participation rate	14/17(82%)	13/17 (71%)	–
Category 1 education provided (%)	4/14(29%)	11/13 (85%)	0.006**
Category 2 education provided (%)	9/14(64%)	13/13(100%)	0.041*
Category 1 or 2 provided (%)	10/14(71%)	13/13(100%)	0.098

**p-value < 0.01, *p-value < 0.05

**Table 2:** Change in number of hours dedicated to concussion-related education (2012 vs 2017)

Medical School	2017 (vs 2012)	
	Category 1 (hrs)	Category2 (hrs)
1	I	I
2	I	I
3	D	I
4	*NR*	*NR*
5	I	I
6	I	I
7	I	I
8	S	I
9	*NR*	*NR*
10	I	I
11	S	I
12	*NR*	*NR*
13	I	D
14	D	D
15	*NR*	*NR*
16	*NR*	*NR*
17	*NR*	*NR*
proportion increase (%)	7/11(64%)	9/11 (82%)
proportion same (%)	2/11(18%)	0/11(0%)
proportion decrease (%)	2/11(18%)	2/11(18%)

*I* Increase in number of dedicated hours, *D* Decrease in number of dedicated hours, *S* Same number of dedicated hours, *NR* Non-responder to either 2012 or 2017 survey

Many participating schools attributed these changes to curriculum oversight committees and renewal processes now recognizing concussion as a common diagnosis and important topic for undifferentiated medical students. Some schools cited increased media attention to the topic and efforts to raise awareness from specialty interest groups as contributing factors. Paradoxically, institutions not offering or limiting concussion-specific teaching deemed the topic not relevant enough for a generalist-oriented curriculum. Another reason mentioned for not prioritizing this topic included lack of related Licentiate of the Medical Council of Canada (LMCC) objectives.

## Discussion

Our 2012 survey showed major deficiencies in concussion education in Canadian medical schools, with only 29% of the responding institutions providing concussion-specific teaching within their curriculum. In contrast, the present follow-up study indicates a considerable improvement with 85% of the schools surveyed offering lectures, case-based learning or seminars on the topic of concussion. Furthermore, 100% of the participating schools now offered at least head injury education sessions that incorporated a concussion component compared to 43% in our initial survey. An increased recognition that concussion and traumatic brain injury are high yield topics for medical students appears to be an important factor driving these changes in curriculum.

Given the rising incidence of concussion and mounting evidence of long term sequelae associated with repeated head trauma, it is crucial that Canadian physicians develop a sufficient knowledge base to reliably diagnose and manage individuals who have suffered a concussion. Despite promising research on the use of advanced neuroimaging and blood-based biomarkers, concussion remains a clinical diagnosis. As such, an in depth understanding of its definition and accurate identification of the common signs and symptoms of concussion are critical to avoid misdiagnosis. Furthermore, the recently published Canadian Guideline for Concussion in Sport underscores the central role that physicians must play in confirming the diagnosis, providing guidance through the return to school and return to play process and granting clearance for return to full activities. Early exposure to the consensus guidelines in medical school—reinforced by exposure to concussed patients in clerkship—are important first steps in ensuring physician competency in concussion assessment and management.

Although we saw a significant improvement in the proportion of Canadian medical schools dedicating time to this topic, it remains worrisome that at least two institutions in Canada still do not provide any concussion-specific teaching. There seems to be an ongoing perception by some schools that concussion education is less relevant to the generalist physician and better suited to residency training programs in emergency medicine or neuroscience-related fields. This is also in line with the results of a recent survey revealing inadequate knowledge surrounding concussion management in a group of Canadian family medicine residents [10]. Although there has been a rise in the number of individuals with mild traumatic brain injury presenting to emergency departments, the majority of these patients are discharged home with instruction to follow up with their primary care provider [11]. As the proportion of emergency department visits for concussive injuries continues to increase, so will the demand on family physicians and pediatricians to become competent in the initial assessment and follow-up of concussed patients, especially in regions where specialist care or multidisciplinary concussion clinic may not be easily accessible. Deferring concussion education to specialist training programs is therefore a potentially risky strategy.

A review of the latest revisions to the Medical Council of Canada’s Objectives for the Qualifying Examinations (MCCQE) also reveal that they still lack concussion-specific learning objectives at the licensure level. This could be another potential explanation for this finding given that medical schools continue to align their curriculum with these objectives. This persistent lack of impetus at the national regulatory level is perplexing given that concussion has now been recognized as a public health priority in Canada and considering the recent mandate by the Prime Minister’s office to develop a Pan-Canadian Concussion Strategy. We recommend revision of the “head trauma” MCCQUE objectives to include clear concussion-specific goals that would at the least require familiarity with the most recent consensus guidelines.

The limitations of this study include the non-response bias inherent in its survey design. Although we informed the schools that all the collected data would be published in an anonymous format, institutions not providing sufficient concussion education may have been less inclined to participate for fear of being criticized. Given the recent shift towards theme-based curriculums and less didactic forms of teaching in undergraduate medical education, it may be more difficult to capture the total number of hours spent on concussion-related learning activities. Furthermore, we cannot ascertain that the participating institutions completed the questionnaires accurately. The number of hours dedicated to concussion and traumatic brain injury during pre-clerkship lectures may also not be a perfect surrogate for the overall adequacy of concussion education during medical school. Indeed, medical students also receive months of supervised training in emergency medicine, surgery, pediatrics and family medicine during their clerkship that provides additional hands-on exposure to concussion patients and foundational knowledge that is essential to those tasked with the medical management of patients presenting with neurological symptoms following head injury. Time allocated to concussion and traumatic brain injury education during these rotations is difficult to estimate and was not assessed in this study. Future studies directly assessing the knowledge and competency of recent medical school graduates in this area could therefore complement our results.

## Conclusions

This follow up survey demonstrates increased exposure to concussion-related learning during undergraduate medical education in Canada between 2012 and 2017. Future studies should assess whether this translates into improved competency in the diagnosis and management of concussion at the residency level and beyond. More in-depth surveys delineating the content of instruction being provided in relation to competency attained upon graduation from medical school are also needed in order to guide optimal curriculum development. The persistent deficiencies present in a minority of schools should be addressed by reiterating the importance of concussion education for undergraduate medical students and by developing clear concussion-specific objectives at the licensure level.

## Appendix

**Table 3:** Summary of Canadian medical school concussion education curriculum

Medical School	Curriculum Description/Format	Teaching hours per category	
		1	2
School 10			
Year 1		0	0
Year 2	Lecture on concussion Lecture on TBI CBL on TBI	0.75	2
Year 3		0	0
Year 4		0	0
Total		0.75	2
School 11			
Year 1		0	0
Year 2	Lecture on TBI CBL on TBI	0	5
Year 3		0	0
Year 4		0	0
Total		0	5
School 13			
Year 1		0	0
Year 2	Lecture on concussion CBL on TBI	1	1
Year 3		0	0
Year 4		0	0
Total		1	1
School 14			
Year 1		0	0
Year 2	Lecture on concussion Seminar on TBI	0.5	0.5
Year 3	TBI - related learning objectives in surgery rotation	0	0
Year 4	Concussion - related learning objectives in emergency medicine rotation	0	0
Total		0.5	0
School 15			
Year 1		0	0
Year 2	Lecture on TBI	0	1
Year 3	TBI and Concussion-related learning objectives in clinical rotations	2	3
Year 4	TBI and Concussion-related learning objectives in clinical rotation	2	3
Total		4	7
School 16			
Year 1		0	0
Year 2	Lecture on TBI	2	3
Year 3		0	0
Year 4		0	0
Total		2	3
Mean		2.65	7.5
Std deviation		2.83	11.02

**Table 4:** School-specific comparison in amount of concussion and TBI-related teaching at 5 year follow -up

Medical School	2012		2017	
	Category 1 (hrs)	Category 2 (hrs)	Category 1 (hrs)	Category 2 (hrs)
1	0	0	3.5	6
2	0	0	2	4
3	0	1	1	10.5
4	0	0	NR	NR
5	2	2	8	6
6	0	1	8	6
7	0	3.5	3	43
8	0	2	0	4
9	3	0	NR	NR
10	0	0	0.75	2
11	0	2	0	5
12	0	2	NR	NR
13	1	6	1	1
14	2	2	0.5	0
15	NR	NR	4	7
16	NR	NR	2	3
17	NR	NR	NR	NR
Response rate	14/17 (82%)		13/17 (76%)	
Mean # of hours (SEM)	0.57	1.54	2.65	7.5

*CBL* Case-based learning, *TBI* Traumatic Brain injury, SEM Standard error of the mean

## Abbreviations

LMCC: Licentiate of the Medical Council of Canada
MCCQE: Medical Council of Canada’s Objectives for the Qualifying Examinations

## References

[1] Gordon KE, Kuhle S (2018). Validation of ‘reported concussion’ within a national health survey. Brain Inj.

[2] Stulemeijer M, van der Werf S, Borm GF, Vos PE (2008). Early prediction of favourable recovery 6 months after mild traumatic brain injury. J Neurol Neurosurg Psychiatry.

[3] Norrie J, Heitger M, Leathem J (2010). Mild traumatic brain injury and fatigue: a prospective longitudinal study. Brain Inj.

[4] Parachute (2017). Canadian Guideline on Concussion in Sport.

[5] Burke MJ, Chundamala J, Tator CH (2012). Deficiencies in concussion education inCanadian medical schools. Can J Neurol Sci.

[6] Donaworth MA, Grandhi RK, Logan K, Gubanich PJ, Myer GD (2016). Is current medical education adequately preparing future physicians to manage concussion: an initial evaluation. Phys Sportsmed.

[7] Tator CH, Davis HS, Dufort PA, Tartaglia MC, Davies KD, Ebraheem A, Hiployee C (2016). Postconcussion syndrome: demographics and predictors in 221 patients. J Neurosurg.

[8] Hiploylee C, Dufort PA, Davis HS, Wennberg RA, Tartaglia MC, Mikuis D, Hazrati LN, Tator CH (2017). Longitudinal study of Postconcussion syndrome: not everyone recovers. J Neurotrauma.

[9] Tartaglia MC, Hazrati LN, Davis KD, Green RE, Wennberg R, Mikulis D, Ezerins LJ, Keightley M, Tator CH (2014). Chronic traumatic encephalopathy and other neurodegenerative proteinopathies. Front Hum Neurosci.

[10] Mann A, Tator CH, Carson JD (2017). Concussion diagnosis and management: knowledge and attitudes of family medicine residents. Can Fam Physician.

[11] Marin JR, Waever MD, Yealy DM, Mannix RC (2014). Trends in visits for traumatic brain injury to emergency departments in the United States. JAMA.

